# HMGA1, Moonlighting Protein Function, and Cellular Real Estate: Location, Location, Location!

**DOI:** 10.3390/biom11091334

**Published:** 2021-09-09

**Authors:** Mireia Pujals, Linda Resar, Josep Villanueva

**Affiliations:** 1 Vall d’Hebron Institute of Oncology (VHIO), 08035 Barcelona, Spain; mpujals@vhio.net; 2 Department of Medicine, Division of Hematology, The Johns Hopkins University School of Medicine, Baltimore, MD 21205, USA; 3 Departments of Medicine (Hematology), Oncology, Pathology and Institute of Cellular Engineering, The Johns Hopkins University School of Medicine, Baltimore, MD 21205, USA; 4 Pathobiology, Cellular and Molecular Medicine and Human Genetics Graduate Programs, The Johns Hopkins University School of Medicine, Baltimore, MD 21205, USA; 5 Centro de Investigación Biomédica en Red de Cáncer (CIBERONC), 28029 Madrid, Spain

**Keywords:** HMGA1, RAGE, unconventional protein secretion, secretome

## Abstract

The gene encoding the High Mobility Group A1 (HMGA1) chromatin remodeling protein is upregulated in diverse cancers where high levels portend adverse clinical outcomes. Until recently, HMGA1 was assumed to be a nuclear protein exerting its role in cancer by transcriptionally modulating gene expression and downstream signaling pathways. However, the discovery of an extracellular HMGA1-RAGE autocrine loop in invasive triple-negative breast cancer (TNBC) cell lines implicates HMGA1 as a “moonlighting protein” with different functions depending upon cellular location. Here, we review the role of HMGA1, not only as a chromatin regulator in cancer and stem cells, but also as a potential secreted factor that drives tumor progression. Prior work found that HMGA1 is secreted from TNBC cell lines where it signals through the receptor for advanced glycation end products (RAGE) to foster phenotypes involved in tumor invasion and metastatic progression. Studies in primary TNBC tumors also suggest that HMGA1 secretion associates with distant metastasis in TNBC. Given the therapeutic potential to target extracellular proteins, further work to confirm this role in other contexts is warranted. Indeed, crosstalk between nuclear and secreted HMGA1 could change our understanding of tumor development and reveal novel therapeutic opportunities relevant to diverse human cancers overexpressing HMGA1.

## 1. Introduction

The diverse functions orchestrated by a cell depend on the action of thousands of proteins working in different cellular compartments at a given time. Until recently, it was thought that most proteins perform one function within a single cellular compartment. However, emerging evidence reveals that an increasing number of proteins break this “one protein-one compartment rule”, thus amplifying the impact that a single protein has on cell fate and behavior [1,2]. Indeed, the term “moonlighting proteins” was established to describe proteins derived from a single gene that perform multiple functions [3]. For example, an enzyme completes a chemical modification based on its specific catalytic site while it performs a different function through protein–protein interactions. A recent study in yeast highlights this concept by revealing that catalytically inactive enzymes can rescue growth. Because the enzymes were engineered to inactivate their catalytic function, these results unveil unexpected functions that are distinct from the enzymatic activity [4]. 

Another class of moonlighting proteins include multi-compartmental proteins that function in a well-established, so-called “canonical compartment” in addition to a previously unknown, “non-canonical compartment” [1,2,5,6]. Shuttling between such compartments could immerse the protein in distinct local environments (pH, redox, cofactors, PTMs) and thereby allow the protein to function differently in each space. Subcellular localization of proteins was presumed to be dictated primarily by the presence of signal peptides that mediate transfer or tropism toward a cellular organelle. Surprisingly, however, the advent of improved technologies for genome-wide proteomics reveal proteins in unexpected locations [2]. The special case of moonlighting proteins with both intra- and extracellular functions indicates that they are secreted proteins, which could include unconventional pathways that allow for their secretion [5,6,7,8]. While many secreted proteins harbor a characteristic N-terminal sequence that allows for entry and secretion via the endoplasmic reticulum (ER)-Golgi apparatus, novel cellular pathways for secreting proteins have been recently discovered, as detailed next [9]. 

Unexpected but exciting results from the laboratory of one of the authors (JV) recently uncovered evidence that the High Mobility Group A1 (HMGA1) chromatin regulator is also secreted from aggressive breast cancer cell lines when cultured in vitro [10]. In this review, we will consider the implications of HMGA1 working in different compartments during tumorigenesis. We will also discuss how the interplay between the nuclear and extracellular functions of HMGA1 could impact cancer biology and possibly normal development.

## 2. Unconventional Protein Secretion (UPS)

Secreted proteins typically contain an N-terminal signal peptide that allows for translocation into the ER. After protein folding, these proteins leave the ER encapsulated in coat protein complex II (COPII)-coated vesicles that link them to the Golgi apparatus. Once they reach the Golgi apparatus, they are sorted and re-encapsulated in vesicles that eventually fuse with the plasma membrane for delivery of vesicular cargo into the extracellular space [11,12]. However, some proteins lacking the N-terminal signal peptide are capable of secretion through unconventional protein secretion (UPS) pathways [13]. The precise molecular mechanisms underlying these pathways are only beginning to emerge, although they appear to be heterogeneous and depend upon the specific secreted protein. Despite these vagaries, a useful classification for such pathways include: (1) direct secretion as soluble proteins, and (2) proteins secreted as extracellular vesicles.

### 2.1. Unconventional Secretion: UPS Pathways (I–IV)

UPS pathways rely on different cellular mechanisms to facilitate the secretion of proteins while bypassing the ER-Golgi apparatus [9]. Type I and Type II UPS pathways are based on the direct translocation of intracellular proteins across the plasma membrane. Proteins that use Type I UPS are self-translocated through the membrane. The Type I UPS is used for example by the Fibroblast growth factor 2 (FGF2) [14,15,16,17] and the HIV trans-activator of transcription (tat) protein. The HIV-tat protein has dual functions, including enhancing transcription of HIV genes in infected cells within the nucleus, and, upregulating inflammatory genes in uninfected neighboring cells as a secreted signaling factor [18]. By contrast, Type II proteins reach the extracellular compartment through ATP-binding cassette transporter (ABC transporters) which secrete only lipidated peptides and proteins. The yeast pheromone a-factor and Schizosaccharomyces pombe m-factor are both exported by the ABC transporter, Mam1 [19]. Type III UPS proteins first enter into the lumen of intracellular vesicles that ultimately fuse with the plasma membrane releasing their cargoes into the extracellular space. In this case, both lysosomes and autophagosomes are used by Type III proteins for secretion. Notable examples of Type III proteins include two acyl coenzyme A—binding proteins: Acba (*Dictyostelium discoideum*) [20] and Acb1 (*Saccharomyces cerevisiae*) [21,22], and interleukin-1β (IL-1β) (Rubartelli et al.). Type III UPS proteins also includes those proteins that are released into the extracellular compartment as vesicles, known as extracellular vesicles (EVs) [23]. Most cells are able to release two types of EVs: exosomes that originate from multivesicular bodies (MVB) [24], and microvesicles generated by exocytosis of the plasma membrane [25]. Finally, a group of transmembrane proteins use the Type IV UPS to reach the plasma membrane bypassing the Golgi apparatus. The best characterized example in this category is the cystic fibrosis transmembrane conductance regulator (CFTR) [26].

### 2.2. Role of Unconventional Secretion in Cancer Biology

Emerging evidence indicates that unconventional secreted proteins participate in diverse biologic processes. However, most relate to responses to cellular stress. From yeast and parasites to mammalian cells, UPS proteins mediate signals from environmental stimuli. In yeast, the redox enzymes detoxifying superoxide radicals, Superoxide Dismutase 1 (SOD1) and Acb1, are secreted upon nutrient starvation [21]. The bacterial parasite, Dictyostelium discoideum, also secretes Acba, an orthologue of Acb1, upon nutrient starvation to mediate spore formation. The human orthologue of SOD1 is also unconventionally secreted from cells using a mechanism similar to that observed in yeast [27]. Mechanical stress has also been linked to UPS. During the embryogenesis of Drosophila melanogaster, for example, mechanical stress upregulates the gene encoding Golgi Re-Assembly and Stacking Protein (GRASP). GRASP mediates the unconventional secretion of aPS1 integrin subunit, which is required in the establishment of the follicular epithelium of the Drosophila developing oocyte [28]. GRASP also mediates the unconventional secretion of Acba [20], suggesting that it may play a wider role orchestrating unconventional protein secretion pathways.

In human biology, inflammation is a common link between UPS and stress. For example, inflammation triggers the secretion of many downstream mediators and unconventionally secreted proteins, including caspase-1 and IL-1β [29,30]. Caspase 1 enzymatically converts the inactive form of IL-1β, pro-IL-1β, to its active form, IL-1β, and increases in IL-1β are associated with aging and diverse cancers. When pathogens or tissue damage induces inflammatory stress, the pattern recognition receptors, such as Toll-like receptors, and RAGE, become activated, after which pro-IL-1β is processed by caspase 1 and secreted through UPS [31,32]. Caspase 1 is also required for the unconventional secretion of cytoplasmic proteins, including galectins, the macrophage migration inhibitory factor (MIF), and FGF2 [29,33]. Recent work highlighting inflammation in fostering aberrant growth and precancerous lesions in diverse tissues also implicates USP proteins in pre-cancerous neoplasia and progression to frank malignancy. The potential role of UPS in tumor biology is further underscored by recent mass spectrometry analyses of secreted proteins which reveal that cancer cells frequently release factors via UPS pathways [2]. While only a handful of UPS proteins have been validated using orthogonal methods, the role of unconventional secretion is emerging as a critical feature of cancer cells that could be modulated in therapy [34,35]. Since metastatic tumor cells encounter varied stressors when leaving their primary site to establish a tumor in another tissue, this could coordinate of myriad of UPS factors, not only at the primary site, but also within the metastatic niches. 

## 3. High Mobility Group Proteins and Cancer

High mobility group (HMG) proteins are a diverse group of basic proteins which are named for their ability to migrate rapidly (thus high mobility) through polyacrylamide gel by virtue of their low molecular weights [36]. HMG proteins are classified into three families: HMGB, HMGN, and HMGA. All HMG proteins are basic, 10–20 kD proteins which harbor an acidic carboxyl terminus and modulate chromatin structure [37,38,39]. Each family, however, is distinguished by unique DNA and/or nucleosome binding motifs. For example, HMGB family members (HMGB1, HMGB2, HMGB3, HMGB4) are defined by 2 HMG-box motifs that mediate binding to DNA without sequence specificity [39,40,41]. They are the most abundant HMG proteins and were the first HMG proteins to be identified as secreted factors where they function in mediating signals involved in sensing injury, DNA damage, immune activation, inflammation, and cancer. HMGB1 can be passively released from necrotic cells by crossing permeabilized membranes once released from the chromatin [42]. HMGB1 is also actively secreted from damaged cells using an autophagy-dependent secretion (Type III UPS pathway) [40,41]. Indeed, knockdown of key autophagy proteins, including ATG5 and ATG7, abrogates the secretion of HMGB1 [43]. Once in the extracellular space, HMGB1 triggers an inflammatory response. HMGN proteins are found only in vertebrates and include five members: HMGN1, HMGN2, HMGN3, HMGN4, HMGN5 [44]. They lack an HMG box, but contain a positively charged, nucleosome-binding “N” domain that mediates specific binding to nucleosomes [45,46]. HMGN proteins are also be released from cells. For example, extracellular HMGN1 functions as an “alarmin“ or factor that activates the immune [47,48]. In contrast to other HMG proteins, HMGA proteins are defined by three AT-hook motifs that mediate binding to the minor groove of B-form DNA at AT-rich regions. This family includes: (1) HMGA1a/HMGA1b isoforms, encoded by the *HMGA1* gene (human chr 6p21) through alternatively spliced mRNA; (2) HMGA1c, the product of a rare splice variant found in testes; (3) HMGA2, a protein highly homologous to HMGA1, but encoded by the *HMGA2* gene (chr 12q15) [49,50]. HMGA proteins lack an HMG box and appear to bind to DNA with sequence specifically [51,52]. 

HMGA proteins are abundant during embryogenesis, but absent or barely detectable postnatally in most differentiated, mature tissues [53]. In embryonic stem cells, HMGA1 induces transcriptional networks involved in self-renewal and pluripotency [54]. HMGA1 becomes aberrantly re-expressed in most aggressive human tumors, where high levels portend poor differentiation status and adverse clinical outcomes [55,56,57,58]. Intriguingly, HMGA1 proteins are among a 13 gene signature encoding transcriptional regulators in embryonic stem cells [59]. Importantly, this signature portends adverse outcomes in brain, bladder, and breast cancers. When overexpressed in lymphoid cells of transgenic mice, Hmga1 induces aggressive leukemia by up-regulating transcriptional networks active in rapidly proliferating stem cells, poorly differentiated cancer cells, and inflammation [60,61]. While mechanisms driving *HMGA1* expression in cancer are incompletely understood, growth factors, cancer-associated mutations, including *Kras* or mutant *Apc*, and oncogenic transcription factors, such as cMYC, up-regulate *HMGA1* in specific contexts, demonstrating that diverse oncogenic pathways converge on *HMGA1* to induce its expression [62,63,64,65,66,67]. In intestinal stem cells, HMGA1 also amplifies Wnt signals from the stroma and epithelial niches by inducing expression of genes encoding both Wnt agonist receptors and Wnt effectors, such as cMyc and Sox9 [68]. Together, these findings suggest that HMGA1 could foster tumor progression through both cell-intrinsic and cell-extrinsic or stromal interactions. As presented next, recent work suggests that HMGA1 is secreted via UPS and could thereby drive tumorigenesis and stem cell properties via direct interactions with the stroma. 

## 4. Extracellular Oncogenic Role of HMGA1

Until recently, HMGA1 was assumed to be a nuclear protein exerting its role in cancer by transcriptionally modulating different signaling pathways. However, the laboratory of one of the authors (JV) recently uncovered exciting evidence implicating HMGA1 in extracellular oncogenic function in triple-negative breast cancer (TNBC) cell lines [10]. The secreted form of HMGA1 was mechanistically linked to its role in tumor invasion and metastasis since blocking the extracellular HMGA1 reduced the metastatic burden in a xenograft model of TNBC. This same study also found that an extracellular localization of HMGA1 in human TNBC primary tumors is associated with the presence of distant metastasis in TNBC patients. 

### 4.1. HMGA1 Secretion and Casein Kinase 2 (CK2)

The Villanueva laboratory discovered that HMGA1 is secreted in a regulated fashion from an invasive TNBC breast cancer cell line (MD-MBA-231) [10]. While secretion did not appear to be mediated by extracellular vesicles, the precise UPS pathway employed in this setting remains unknown. However, CK2 was implicated in HMGA1 secretion in these studies. CK2 is known to phosphorylate three serine residues (99, 102, and 103) at the C-terminal region of HMGA1 [69]. In invasive TNBC cells, either inhibiting CK2 or mutating the three HMGA1 serines phosphorylated by CK2 resulting in decreased HMGA1 secretion [10]. Notably, CK2 is linked to the unconventional secretion of other intracellular proteins, including the HMGB1 protein [70]. This study also showed that inhibiting CK2 pharmacologically disrupts invasion in a TNBC cell line. Further, mutation of the three serines (99, 102, 103) in the HMGA1 C-terminus disrupts invasive properties of the TNBC cell line. Intriguingly, CK2 inhibitors were also found to decrease tumor cell invasion in glioblastoma cell lines (GBM) [71,72,73,74]. A recent study using organotypic GBM experimental models also found that the transcriptional repressor interferon regulatory factor 3 (IRF3) decreases invasion in GBM cells [71]. Because CK2 negatively regulates IRF3, inhibiting CK2 up-regulates IRF3, and suppresses invasiveness in GBM cells [71]. Another study showed that either silencing *HMGA1* expression or mutation of a CK2 phosphorylation site—Serine 102—of HMGA1 enhanced efficacy of gefitinib in resistant NSCLC cells through reactivation of the downstream signaling of EGFR [75]. However, it remains unknown whether extracellular HMGA1 contributed to drug resistance in cells. 

### 4.2. Receptor for Advanced Glycation End Products (RAGE)

Results from the Villanueva study indicate that HMGA1 mediates its extracellular signaling via the receptor for advanced glycation end products (RAGE receptor) by acting as a ligand [10]. This effect is pERK-dependent and results in enhancing migration, invasion, and metastasis. RAGE is a mammalian pattern recognition receptor from the immunoglobulin superfamily, which is highly expressed during embryogenesis, but rarely expressed in adult healthy tissue [76,77]. The exception is the lung, where RAGE is constitutively expressed in alveolar cells. In the lung, RAGE fosters interactions between adjacent cells, alveolar epithelial cells, and the basal membrane [78]. The protein level of RAGE is tightly regulated by the presence of its ligands, which include advanced glycation end-products (AGEs), proteins of the S100 family, fibrillar proteins, such as amyloid-beta, and HMGB1. More recent data suggests that HMGA1 also regulates RAGE activity and protein levels. Because multiple ligands interact with RAGE, which in turn, signals through diverse pathways, the downstream cellular consequences of RAGE activation are complex. Upon ligand binding to the V domain of RAGE, the receptor oligomerizes, which allows the interaction with different intracellular effectors including Diaphanous 1 (mDIA) [77,79,80]. Activation of RAGE modulates diverse signaling pathways, including nuclear factor kappa-light-chain-enhancer of activated B cells (NF-kB), mitogen-activated protein kinase (MAPK), the Janus kinase (JAK)-signal transducer, and activator of transcription (STAT) [77,79].

**Figure 1:**
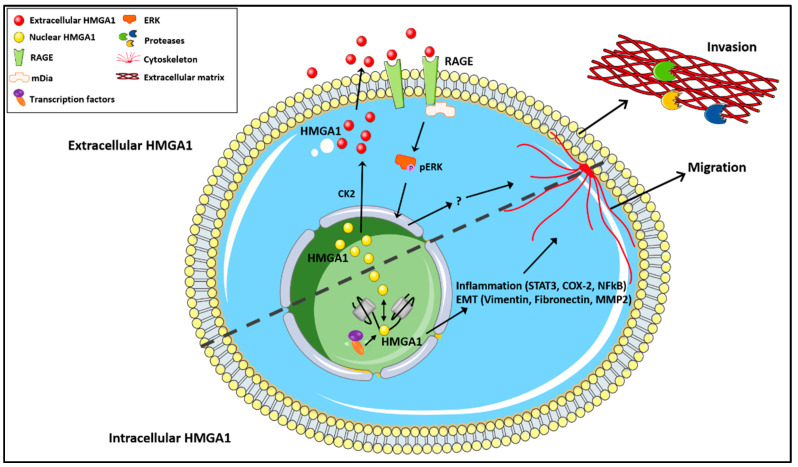
HMGA1 was recently discovered in different cellular compartments during tumorigenesis. The scheme illustrates known molecular mechanisms that mediate HMGA1 function in cancer.

Despite the broad array of ligands and pathways regulated, most of the cellular effects induced by RAGE are linked to inflammation [80,81]. RAGE is involved in the resolution of acute inflammation in diverse settings, including wound healing, immune adaptative responses, and nerve regeneration [82]. However, sustained activation of RAGE is directly linked to chronic inflammation, and mediates deleterious effects in common aging-associated diseases such as diabetes, neurodegenerative diseases, and arthritis [77,80]. Although RAGE has not been extensively studied in cancer, many RAGE ligands, such as S100 proteins, are known to foster tumor growth and metastatic progression [10,83,84,85]. Furthermore, increasing evidence underscores the role of chronic inflammation tumor initiation beyond the intestinal tract and colon [86]. Together, these findings implicate RAGE in cancer and suggest that an HMGA1-RAGE autocrine loop could contribute to tumor progression (Figure 1). This is particularly exciting since receptors and secreted factors can be targeted in therapy more readily than most transcription factors. 

## 5. Reconciling HMGA1 Functions across Compartments

The existence of an active, secreted HMGA1 fundamentally changes the current paradigm for HMGA1 function in tumorigenesis. Given the promise of targeting secreted factors, this intriguing discovery warrants further research. Based on existing knowledge, we discuss the potential consequences of cross-talk between the nuclear and extracellular HMGA1 in cancer biology.

### 5.1. Integrating the Inflammatory Response

HMGA1 is linked to inflammatory processes in diverse contexts [61]. In diverse solid tumors and hematologic malignancy, HMGA1 directly induces expression of genes involved in mediating inflammatory processes. For example, as published by one of the authors (LR) and many others, HMGA1 directly induces expression not only of inflammatory mediators, such as interferons, cytokines, and chemokines, but also of receptors that activate inflammatory responses. HMGA1 was first shown to induce expression of *Interferon-β* (*IFNβ*) by recruiting an enhanceosome to key regulatory regions upstream of the coding region, and other cytokines and inflammatory mediators were subsequently linked to HMGA1 transactivation. In *Hmga1* transgenic mice, for example, Hmga1 directly induces the *Signal Transduction and Activator of Transcription 3* (*STAT3)* gene with leukemic transformation [87]. STAT3 functions in inflammation, angiogenesis, and tumor progression, and both *HMGA1* and *STAT3* are up-regulated and co-expressed in hematologic malignancy [88]. Of note, JAK inhibitors are used clinically in hematologic malignancy, although it is not clear whether they alter HMGA1 function [89]. Gene expression studies from the transgenic lymphoid tumor model and other models revealed diverse genes involved in inflammation. 

The female *Hmga1* transgenic mice also develop uterine tumors, in part, by inducing the gene encoding cyclo-oxygenase-2 (COX2). Indeed, COX2 inhibitors (COXibs) repressed uterine tumor growth in the *Hmga1* females and in xenografts of human uterine sarcomas in immunosuppressed mice [90]. HMGA1 and *COX2* are also up-regulated and co-expressed in human uterine sarcomas (leiomyosarcomas) [91] in addition to pancreatic tumors [92]. Like STAT3, COX2 is a pleiotropic factor involved in tumor initiation, progression, and inflammatory signaling [49] (Figure 1) and COX-2 inhibitors decrease the incidence of gastrointestinal tumors in mice and humans as well as a subset of tumors arising in other tissues. Intriguingly, HMGA1 was first linked to *COX2* expression in vascular endothelium in the setting of hypoxia [93] suggesting that this pathway may function in diverse settings.

Inflammatory mediators also induce *HMGA1* expression, such as viral infection, or lipopolysaccharide (LPS), which also activate other inflammatory cytokines. HMGA1 could therefore serve as a hub and feed-forward loop to enforce *HMGA1* overexpression and amplify inflammatory signals. Following viral infection or LPS, for example, HMGA1 orchestrates the assembly of NF-κB subunits, p50 and p65, to the *IFNβ* promoter, inducing *IFNβ* expression and secretion [61]. Intriguingly, many regulatory regions of HMGA1 transcriptional targets harbor NF-κB binding sites, suggesting that these factors frequently collaborate to modulate gene expression. Because NF-κB is a well-established regulator of inflammation, this interaction further links HMGA1 to inflammatory signals. HMGA1 also modulates the inflammatory response following acute lung injury by facilitating the binding of NF-κB and TNF-α to regulatory elements of the *E-selectin* and *P-selectin* genes [94]. Selectins are adhesion molecules that promote transmigration of leukocytes across the endothelial surface into tissues. Studies with *selectin* genes showed that blocking DNA binding by HMGA1 disrupts recruitment of NF-κB to *selectin* promoters, and thereby decreases the inflammatory response. Based on the link between inflammation and cancer, HMGA1- NF-κB complexes, also known as the HMGA1 enhanceosome, are likely to contribute neoplastic transformation [61]. 

While intranuclear HMGA1 contributes to inflammation by modulating expression of cytokines, chemokines, and their receptors, the existence of extracellular-HMGA1 (eHMGA1) opens many avenues for further HMGA1 interactions that could drive both inflammation and cancer. Since eHMGA1 functions through RAGE, and RAGE signals through NF-κB, this provides yet another mechanism whereby HMGA1 could amplify NF-kB inflammatory cytokines. While it is not yet clear whether eHMGA1 occurs more broadly in human cancer and activates NF-κB through RAGE, it should be studied further given the immense clinical implications. 

### 5.2. Epithelial-to-Mesenchymal Plasticity (EMP)

HMGA1 also regulates genes involved in plasticity and an epithelial-mesenchymal transitions. Since EMT is not a binary (E/M) process but involves multiple partial states that give rise to a high degree of cell state plasticity, Epithelial-Mesenchymal plasticity (EMP) [95] is a more accurate definition of the process previously known as EMT. From development to wound healing and acute inflammation, EMP is essential for life [96]. However, during tumor initiation, progression, and cancer therapy, tumor cells hijack EMP networks to thrive [97]. Prior studies found that HMGA1 regulates mesenchymal genes in colon cancer cells, such as Vimentin (VIM), N-cadherin (CDH2), E-cadherin (CDH1), and Fibronectin (FN1) [98,99,100,101]. This is of interest, since both nuclear and eHMGA1 could collaborate in promoting epithelial-to-mesenchymal plasticity (EMP). Prior work from diverse models, including TNBC, indicate that tumor cells hijack EMP networks to thrive during tumor initiation, progression, and cancer therapy [97]. TNBC cells, where extracellular HMGA1 was first described, are characterized by a de-differentiated, mesenchymal like state, which appears to foster tumor initiation and progression. Indeed, those tumor cells with more mesenchymal features frequently localize to the tumor invasive front [102,103]. Thus, regulators of EMP genes appear to play fundamental roles in tumor progression and development and both intranuclear and eHMGA1 could function in these pathways. 

In addition to cancer, EMP plays a key role in embryogenesis. During embryonic development, cellular plasticity is required to generate the specialized cell types that will constitute tissues and organs. Morphogenesis occurring during embryogenesis relies on the dynamic interconversion between epithelial and mesenchymal cells, i.e., EMP. HMGA1 is widely expressed during embryogenesis in different tissues, and its loss causes developmental abnormalities. For example, a recent paper found that HMGA1 is required for neural crest formation [104]. Neural crest cells are a population of cells from the neuroectoderm, originally derived from the embryonic ectoderm, which undergo EMP as they acquire mesenchymal properties. These cells are highly migratory and, after delamination, they migrate and generate different specific cell types [105]. HMGA1 is first involved in the specification of the neural plate border through the action of Pax7. Second, HMGA1 modulates Wnt signaling pathways and the Slug transcription factor to foster cranial neural crest emigration and migration. Postnatally, neural crest-derived cells can undergo oncogenic transformation, and not surprisingly, *HMGA1* is overexpressed in at least a subset of tumors derived from neural crest cells. Thus, HMGA1 may induce EMP signals in a tightly regulated fashion during development, while this process becomes de-regulated during tumorigenesis.

Because HMGA1 is linked to EMP in cancer and development, it is plausible that crosstalk between the nuclear and the extracellular forms of HMGA1 occur with plasticity. One potential multitasking protein, NUMB [106], could also participate in maintaining plasticity together with HMGA1. NUMB is expressed by neural progenitor cells, neuroblasts, and stem cells where it is asymmetrically segregated in cells. This asymmetry allows the daughter cells containing Numb to acquire a different cell state than the daughter cells lacking it [107]. A recent study also found that HMGA1 downregulates NUMB in brain tumor stem cells, which could maintain the tumor cells in a more de-differentiated, stem-like state and contribute to tumor progression. Another recent study showed that the mice deficient in RAGE develop defective muscle regeneration due to defective myoblast cell division [108]. These correlative findings suggest that NUMB, RAGE, nuclear HMGA1, and extracellular HMGA1 could cooperate in regulating stem cell fate and plasticity. 

Because RAGE may mediate crosstalk between nuclear and eHMGA1 with inflammation, this interaction could also participate in EMP. Indeed, RAGE is linked to EMP in tumor cells originating from different tissues. In breast and lung cancer cells, for example, lysophosphatidic acid (LPA) drives EMP through its interaction with RAGE [109]. Another study implicated RAGE and the NF-kB pathway in EMP in breast cancer [110]. In this study, miR-185-5p was overexpressed and served to repress RAGE, thereby reversing EMP properties in MDA-MB-231 cells [111]. In the prostate cancer cell line, PC-3, silencing *RAGE* also repressed EMT markers, including matrix metalloproteases, disrupting cell migration and invasion [112]. RAGE is also involved in fibrotic diseases marked by aberrant EMP, such as nasal polyp formation [113]. Another recent study showed that the HMGB1-RAGE signaling mediates EMP in upper airway epithelial cells [114]. In the renal tubular cells, another study found that release of HMGB1 up-regulated connective tissue growth factor (CTGF) and transforming growth factor β (TGFβ) to drive renal fibrosis in a RAGE-dependent fashion [115]. Thus, RAGE functions not only in development, inflammation and cancer, but also in fibrosis.

## 6. Conclusions and Future Directions

The discovery of an extracellular HMGA1-RAGE autocrine loop in invasive TNBC cell lines implicates HMGA1 as a moonlighting protein with different functions depending upon cellular location. Prior studies of HMGA1 in cancer and development focused on its role in the nucleus as a regulator of chromatin structure and function. Indeed, HMGA1 proteins have been described as architectural transcription factors for this reason. Because *HMGA1* is overexpressed in most aggressive tumors where it functions in tumor progression, further study of its potential roles in the extracellular space promise to provide important insight relevant to diverse tumors. The unconventional secretion of HMGA1 warrants further study since this location provides an exciting avenue for therapeutic targeting. If secreted HMGA1 is a common feature in settings beyond TNBC cells, this could have important implications in cancer biology [35]. 

**Figure 2:**
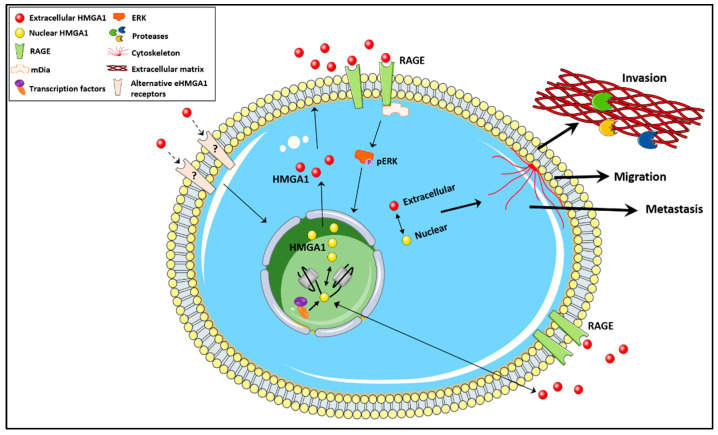
The unconventional secretion of HMGA1 warrants further studies to understand the role of extracellular HMGA1 in cancer biology. Future research should answer questions related to: the molecular mechanism driving the subcellular location of HMGA1, the factors controlling HMGA1 secretion, whether eHMGA1 mediates an oncogenic function by binding to receptors other than RAGE, whether there is a cross-talk between the nuclear and extracellular HMGA1, and how the equilibrium between the two forms of HMGA1 dictates the role of HMGA1 in cancer biology.

Key research questions that remain unanswered include the following (see Figure 2):

(1) What factors dictate the location of HMGA1?
(2) Are posttranslational modifications involved (similar to those described for HMGB1)?
(3) What triggers secretion together with CK2 or independent of CK2?
(4) Which UPS pathway leads to HMGA1 secretion?
(5) Do receptors other than RAGE mediate eHMGA1 function?
(6) How does intranuclear HMGA1 collaborate with eHMGA1?
(7) Does eHMGA1 function in embryonic development
(8) Is HMGA1 secreted in vivo?
(9) Do diverse tumors secrete HMGA1?

In closing, the prospect that HMGA1 functions in the extracellular space by signaling through RAGE and possibly other cell surface receptors opens many exciting avenues for both future study and therapeutic targeting. Further elucidation of secreted proteins and their function in noncanonical settings should provide insight relevant to normal growth and development in addition to what goes awry with neoplastic transformation and tumor progression. Similar to real estate investments, a key feature of moonlighting protein function is location, location, location!
